# Role of individual perceptions in the consistent use of malaria preventive measures: mixed methods evidence from rural Rwanda

**DOI:** 10.1186/s12936-019-2904-x

**Published:** 2019-08-08

**Authors:** Domina Asingizwe, P. Marijn Poortvliet, Constantianus J. M. Koenraadt, Arnold J. H. van Vliet, Chantal Marie Ingabire, Leon Mutesa, Cees Leeuwis

**Affiliations:** 1 College of Medicine and Health Sciences, University of Rwanda, Kigali, Rwanda; 2 Strategic Communication Group, Wageningen University and Research, Wageningen, The Netherlands; 3 Laboratory of Entomology, Wageningen University and Research, Wageningen, The Netherlands; 4 Environmental Systems Analysis Group, Wageningen University and Research, Wageningen, The Netherlands; 5 Knowledge, Technology and Innovation Group, Wageningen University and Research, Wageningen, The Netherlands

**Keywords:** Risk perception, Perceived efficacy, Malaria prevention, Bed net use, Rwanda

## Abstract

**Background:**

Malaria preventive measures, including long-lasting insecticide-treated bet nets (LLINs), indoor residual spraying (IRS), and controlling mosquito breeding sites, are key measures to achieve malaria elimination. Still, compliance with these recommended measures remains a major challenge. By applying a novel and comprehensive model for determinants of malaria prevention behaviour, this study tests how individual perceptions influence the intentions to use malaria preventive measures and explores strategies that stimulate their consistent use.

**Methods:**

The study was carried out in the sectors of Ruhuha and Busoro, Rwanda during October and November 2017, and these were conducted into two phases. Phase one involved a questionnaire survey (N = 742), whereas Phase two employed a qualitative approach that included nine focus group discussions, seven key informant interviews, and three in-depth interviews.

**Results:**

The findings of the quantitative study showed that participants very often use LLINs (66.6%), accept IRS (73.9%), and drain stagnant water in case of presence (62%). The intentions to use malaria preventive measures were consistently driven by perceived severity, perceived self-efficacy, perceived response efficacy, and subjective norms, and hindered by perceived barriers. The intentions were also positively associated with the actual use of LLINs, acceptance of IRS, and drainage of stagnant water. There is no evidence that either not having enough LLINs (ownership of at least one bed net in the household, here referred to as availability) or having sufficient LLINs (having one LLIN per two people in the household, here referred to as accessibility) moderated the relationship between behavioural intentions and actual use of LLINs. The qualitative study indicated that participants believed malaria risk to be high and perceived a high mosquito density. They also believed that repetitive malaria episodes are caused by the perceived low effectiveness of anti-malaria medications. Lack of LLINs increased the perceived added value of LLINs, and together with the increased malaria burden increased the perceived response efficacy. Participants highlighted the need to continuously mobilize and engage community members especially those who do not use LLINs when having one, and those who do not accept the spraying activities.

**Conclusion:**

Malaria prevention interventions should target individual perceptions to enhance consistent use of malaria preventive measures. Three strategies to improve consistent use and acceptance of these measures are highlighted: (1) ensure access to LLINs and regular spraying activities, (2) community mobilization and (3) citizen engagement in malaria prevention activities.

## Background

Malaria, a vector-borne disease, is a serious threat worldwide [1]. From 2010, a reduction in malaria incidence was observed, but around 2014 the rate of decrease halted and even reversed in some countries [1]. One of these countries is Rwanda where since 2011 a significant increase in malaria incidence was observed [2]. Despite this resurgence, significant progress in scaling up of malaria preventive measures is currently taking place [3]. These measures include long-lasting insecticide-treated bet nets (LLINs), indoor residual spraying (IRS), and the control of mosquito breeding sites. The World Health Organization (WHO) recommends using LLINs for all people who are at risk of getting malaria [3]. According to the Rwanda malaria contingency plan 2016–2020, by 2020, 90% of the population at risk should be protected by the following locally appropriate malaria preventive measures (use LLINs, accept IRS, and control all mosquito breeding sites), and 75% should use LLINs consistently [3].

Despite this ambition to increase the consistent use of preventive measures, the latest Rwanda Demographic and Health Survey conducted in 2014–2015 indicated that 81% of households own at least one LLIN, and that only 43% of households have at least one LLIN for every two people [4]. This indicates that the proportion of households with sufficient LLINs (one LLIN for every two people) remains inadequate. The same report indicated that 62% of the visited household members slept under LLINs the night before the survey [4]. Apart from the use of LLINs, the implementation of IRS even dropped in Rwanda due to limited resources [3].

Whether people use LLINs consistently, accept IRS, and control mosquito breeding sites depends on many factors including the accessibility to LLINs, and IRS, but also importantly on people’s perception of malaria risk and of the effectiveness of malaria preventive measures [5]. Hence, there is a need to understand people’s perceptions and determine to what extent they predict the intentions towards the actual and consistent use of malaria preventive measures.

Although limited availability of LLINs (not having one LLIN per two people) is a critical factor of non-use [6, 7], several studies indicate that around 20% of the people who own LLINs still do not use them [8–12]. This calculation of use gap was mainly based on two indicators: ownership of at least one LLIN, and population use of LLIN, and this calculation may be misleading since the consideration of ownership of at least one LLIN may leave out those with insufficient LLINs (availability) [13]. However, the extent to which both ownership of at least one LLIN and accessibility (having one bed net per two people in the household) affect the consistent use of LLINs is still unclear. Other studies reported IRS refusal and failing to remove stagnant water bodies [14–16]. From these studies, there are indications that the use of malaria preventive measures is partly determined by the perception of the risk of malaria infection and the effectiveness of these measures, by the increased prevalence of insects in the house after spraying, and by throwing LLINs away once they become dirty or torn out [5, 14–19].

Only few studies assessed the determinants of consistent bed net use [5, 20], and as of yet, there is no evidence of the extent to which the identified determinants affect the intentions to use malaria preventive measures consistently, how important they are and how they can be influenced. In addition, a study using an integrated model of determinants of malaria preventive behaviour [21] that maps the association between these perceptions and intentions to use these preventive measures is lacking. The current study aims to assess how individual perceptions influence the intentions to use malaria preventive measures and to explore strategies that may stimulate consistent use of malaria preventive measures. Therefore, the study addressed the following research questions: (1) how do community members perceive the risk of malaria? (2) how do community members perceive the effectiveness of malaria preventive measures? (3) to what extent do individual perceptions influence the intentions to use malaria preventive measures? and (4) what strategies can, according to community members, be used to stimulate consistent use of malaria preventive measures? In this paper, “availability is defined as having at least one LLIN and this is considered as not having enough LLINs” as most of households have more than two people. In addition, “accessibility is defined as having one LLIN per two people and this means having sufficient LLINs in the household”. Both variables (availability and accessibility) were included in the conceptual framework and statistical models as moderators between behaviour intentions and consistent use of LLINs.

## Conceptual framework

**Fig. 1:**
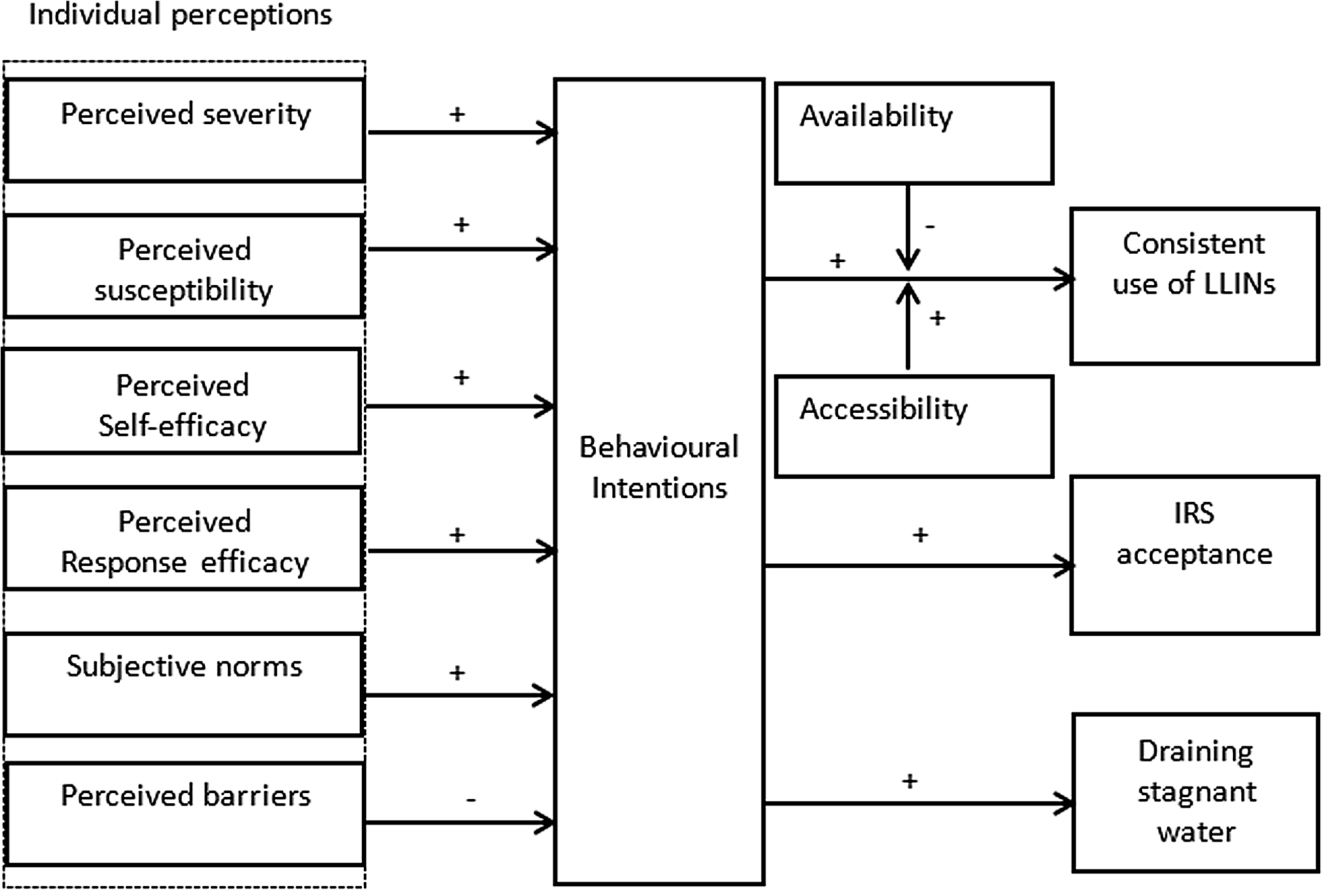
Overview of how determinants influence the use of malaria preventive measures [21]. This figure describes how the conceptual model’s determinants positively or negatively predict intentions to consistently use malaria preventive measures. It points out different hypotheses related to how perceived severity of malaria, perceived susceptibility, perceived self-efficacy, perceived effectiveness of malaria preventive measures, subjective norms, and perceived barriers influence intentions to use malaria preventive measures. In addition it illustrates how availability and accessibility moderate the effect between intentions and actual use of LLINs; and the relationship between behavioural intentions and actual use of LLINs, acceptance of IRS, and draining of stagnant water

In order to understand how individual perceptions influence behavioural intentions to use malaria preventive measures, the study uses an integrated model of determinants of malaria prevention behaviour [21]. The model categorizes individual perceptions into perceived severity, perceived susceptibility, perceived self-efficacy, perceived response efficacy, subjective norms, and perceived barriers [21]. Below, the rationale behind the different determinants in the model (see Fig. 1), and how the model’s determinants positively or negatively predict intentions to consistently use malaria preventive measures are described.

Malaria risk perceptions have been reported to predict consistent use of LLINs [19, 20]. If people intend to use malaria preventive measures because of associated high-perceived risk of malaria, then it is likely that once the malaria prevalence reduces, the adoption rate of these measures also decreases [20]. Some people plan to use the LLINs because they have noted how they prevent malaria and think that consistently using them is better to remain free from malaria even if the prevalence of malaria reduces. However, for other people the usage of LLINs drops because people think that malaria is no longer a problem [20]. Thus, perceived severity of malaria positively influences intentions to use and accept malaria preventive measures. People who do not perceive themselves to be at risk of having malaria are less likely to adopt these measures even if they own them [22]. Consequently, the perceived susceptibility is positively associated with the behavioural intentions to use malaria preventive measures (see Fig. 1).

Some studies documented that the perceived self-efficacy (belief in one’s ability to use malaria preventive measures), and the perceived response efficacy (people’s beliefs about the effectiveness of malaria preventive measures) influence the consistent use of these measures [5, 18]. Similarly, Beer et al. [23] indicated that perceived response efficacy of LLINs remains an important reason for using them in case of a reduction in malaria incidence and associated low malaria risk perception. Accordingly, both perceived self-efficacy and response efficacy will positively influence the intentions for the consistent use of malaria preventive measures (see Fig. 1). Among other factors that influence intentions to use LLINs consistently, Koenker et al. [20] presents the role of subjective norms. If a high proportion of people in the community sleep under LLINs, accept IRS, and drain stagnant water, then many people in that community may intend to follow the apparent social norm and may plan to consistently do the same [5]. Thus, subjective norms and behavioural intentions are expected to have a positive relationship (Fig. 1). However, when people believe that the chemicals in LLINs are no longer effective in killing mosquitoes, they are more likely to use them for other purposes [17].

Perceived barriers to use LLINs and accept IRS, including feeling too hot when sleeping under the LLINs (especially in the dry season), discomfort, irritability, and presence of bed bugs or other insects after spraying, were reported in previous studies [6, 16]. These factors were reported to hinder the use and acceptance of malaria preventive measures [6, 16]. Consequently, perceived barriers will negatively influence behavioural intentions (Fig. 1). Previous studies indicated that both ownership of at least one LLIN and access (having sufficient LLINs) are strong determinants for its use [6, 7]. Therefore, not having enough LLINs (having at least one LLIN) and access (having enough LLINs: one per two people) will moderate the effect of intentions on use of LLINs (Fig. 1).

## Quantitative and qualitative approaches to study individual perceptions

**Fig. 2:**
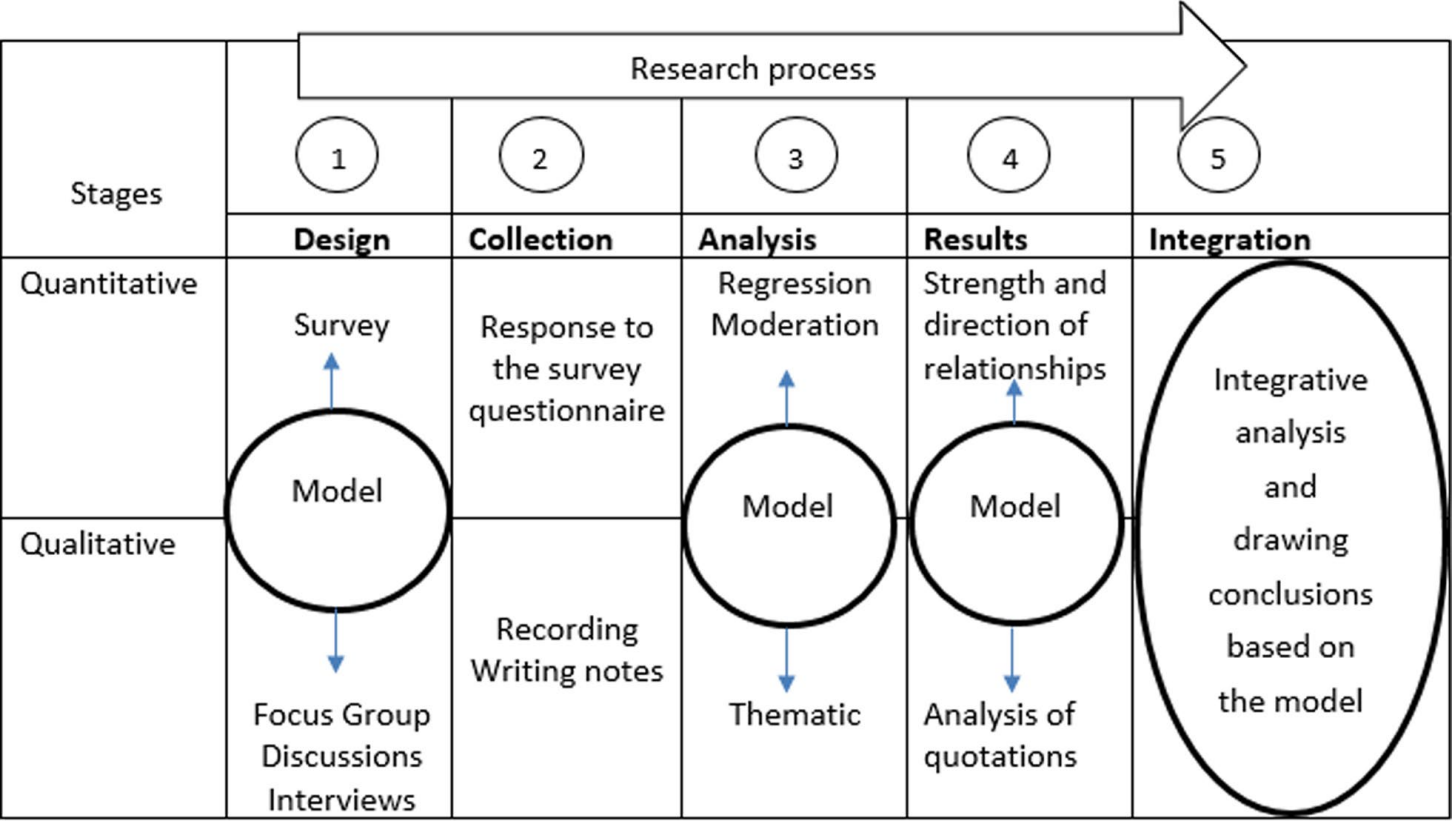
Flow and exchange between the two research phases (quantitative and qualitative). This figure indicates the flow and exchange between the research phases from the research design to its implementation. It also explains how the justice between the two approaches was made, how they complement each other, as well as how the integration between the two research phases was done

The research consisted of two phases (Fig. 2) and these were driven by the research questions which were informed by the conceptual framework. Phase one involved a quantitative study to map and assess the strength and direction of the relationships between the model variables. Phase two involved a qualitative study and was added to enable more comprehensive explanations for why participants have certain perceptions related to malaria and malaria preventive measures, and to identify strategies to enhance the consistent use of these measures. Additionally, the qualitative approach tried to better understand the relationships that were identified in the quantitative phase. Given an equal emphasis among the two phases, the data were collected close together in time. The two phases are linked in that all stages (that is, design, data collection, analysis, results interpretation, and integration) (see Fig. 2) are developed based on the concepts of the research model (conceptual framework). Thus, the research model (called “Model” in Fig. 2), informed all stages of the research process. Consequently, quantitative and qualitative phases complement each other, with the ultimate aim of enabling drawing a robust conclusion. As such, this mixed methods design provides more insights related to the directions and strengths of the relationships indicated in the model and offers the underlying explanations to why people have these perceptions.

## Quantitative phase

### Methods

#### Study setting

The relationships between individual perceptions and behavioural intentions, and the possible moderating role of both not having enough LLINs and having sufficient LLINs on intentions and LLINs use were tested in Ruhuha, a sector in Rwanda’s Eastern province, and Busoro, a neighbouring sector, but in Rwanda’s Southern province. Ruhuha has around 35 villages grouped in five cells, and approximately 5000 households [24], while Busoro has around 40 villages groups in six cells, and approximately 8000 households. Both sectors have numerous marshlands and water streams draining into the Akagera River, and were selected as they are among the highest malaria-endemic areas in the country due to the presence of these marshlands and various rice farming activities that are a source for malaria vectors [24].

#### Study population and sample size

The study population included all households in Ruhuha and Busoro sectors. A sample of 742 households with equal numbers from each sector was selected. To choose this sample, a multistage sampling method was used, including stratified random sampling. Each cell within each sector was considered as a stratum, giving a total of 11 strata. Cells have a range of five to nine villages. At the cell level, two villages were selected by a simple random sampling. At the village level, lists of households were provided by the village leaders, and a systematic random sampling was used to draw a sample of households to be visited. The nth household was determined based on the size of the village, and a starting number was chosen randomly.

### Data collection

At the start, an initial meeting with the village leaders of selected villages was conducted. The selected households were notified ahead of time by the respective village leaders. A team of six research assistants conducted the survey. The team was fluent in the local language (Kinyarwanda) and the research assistants received a 1-day training session to interview the respondents. The questionnaire was installed on Samsung Galaxy 2 Tablets using Open Data Kit (ODK) software. A written informed consent was signed by the head of a household prior to initiation of the data collection. Whenever possible and with participants’ agreement, direct observations were made to minimize self-reported biases, mostly in the case of LLIN use. Participants did not receive any form of compensation or reimbursement.

### Variables

The questionnaire was first developed in English, translated in Kinyarwanda, back-translated into English, and then pre-tested in a pilot study of 10 households selected randomly in the Mareba sector, Eastern Province, Rwanda. All translations were made by professional translators including members of the project team and cross-checked by native speakers. The translators were asked to review and cross check the items and identify any problems in language, terminology, understandability, and relevance.

All independent variables were measured on five-point Likert scales (from 1—strongly disagree to 5—strongly agree). These included perceived severity, perceived susceptibility, perceived self-efficacy, perceived response efficacy, subjective norms, and perceived barriers. The latter, was assessed into two categories: perceived discomfort and perceived lack of information. Except behavioural intentions, which was measured on five-point Likert scales (from 1—strongly disagree to 5—strongly agree), other dependent variables including consistent use of LLINs, acceptance of IRS, and draining stagnant water were measured on five-point Likert scales (from 1 almost never to 5-very often).

### Data analysis

SPSS version 21 was used to test the relationships between the model variables. Descriptive statistics using frequencies, percentages, means and standard deviations were used to summarize the data. Cronbach’s alpha values were calculated to determine the internal reliability of the variables of interest. Correlation coefficients were computed to explore the relationships between the predictor variables. The mean item scores were obtained by dividing the sum scores by the number of items for each subscale. To determine the independent predictors of the behavioural intentions and malaria preventive measures, a hierarchical linear regression analysis was conducted consisting of two steps. In the first step, demographic characteristics (age, gender, and education) were included as control variables. In step 2, the predictor variables perceived severity, perceived susceptibility, perceived self-efficacy, perceived response efficacy, subjective norms, perceived discomfort, and perceived lack of information were added. To investigate whether the strength and direction of the relationship between behavioural intentions and use of LLINs could be changed by the two moderators availability and accessibility of LLINs, a moderation analysis was conducted. This was done through assessing the statistical significance of interaction terms from: (1) availability (not having enough LLINs and defined as having at least one LLINs), and (2) accessibility (defined as having sufficient LLINs: one per two people) and behavioural intentions in the regression model. The two moderators were included to explore whether: (1) having at least one LLIN will improve their consistent use by buying others in case of need, and (2) having sufficient LLINs will motivate people to increase their consistent use of them. As used in other studies, the access (having one LLIN per two people) was calculated by dividing the number of LLINs owned by each household by the number of household members [who slept in that household the previous night preceding the survey] [13, 25].

## Results

### Participants characteristics

Of the 742 study participants, 59% were female, more than half had either no formal education or incomplete primary school, and most of them were farmers (78.6%) (Table 1). The average age of the respondents was 43.3 years (SD = 14.8), and the majority (69.4%) owned a bed net.

**Table 1:** Characteristics of the study participants

Variables	Variable categories	n (%)
Gender	Male	304 (41)
	Female	438 (59)
Education	None	247 (33.3)
	Incomplete primary	248 (33.4)
	Complete primary	183 (24.7)
	Incomplete secondary	32 (4.3)
	Complete secondary	28 (3.8)
	Tertiary	4 (.5)
Occupation	Farmer	583 (78.6)
	Public servant	5 (.7)
	Self-employed	40 (5.4)
	Private servant	15 (2)
	Student	5 (.7)
	Unemployed	94 (12.7)
Age	Mean (SD)	43.3 (14.8)
N of HH members	Mean (SD)	4.9 (2.2)
N of sleeping rooms	Mean (SD)	2.5 (1.1)
N of beds	Mean (SD)	2.2 (1.0)
LLIN ownership	No	227 (30.6)
	Yes	515 (69.4)
LLIN used last night (among those who own them)	No	63 (12.2)
	Yes	452 (87.8)
Household members that use bed net last night	Every household member	310 (68.6)
	Only adults	70 (15.5)
	Only few people (mixture group)	42 (9.3)
	Only children	30 (6.6)
N LLINs owned	Mean (SD)	2.01 (1.13)
Access (one LLIN per two people)	Mean (SD)	.47 (.34)
Ever heard about IRS	No	56 (7.5)
	Yes	686 (92.5)
Presence of stagnant water	No	585 (78.8)
	Yes	157 (21.2)
Presence of bed bugs	No	297 (40)
	Yes	445 (60)

### Internal reliability and correlations between predictor variables

As shown in Table 2, the Cronbach’s alpha values for eight constructs ranged from .64 to .90 indicating that the scales used had adequate reliability. The behavioural intentions as the main predictor variable was significantly positively associated with perceived self-efficacy and response efficacy, and negatively associated with perceived discomfort and lack of information. All relationships will be fully explored in the following analysis.

**Table 2:** Bi-variate correlations between study variables

Subscales	N of items	Cronbach’s alpha	*M (SD)*	1	2	3	4	5	6	7
1. Perceived severity	8	.76	4.38 (.51)							
2. Perceived susceptibility	8	.71	3.44 (.68)	.24**						
3. Perceived self-efficacy	7	.77	4.37 (.50)	.42**	.11*					
4. Perceived response efficacy	6	.64	3.92 (.63)	.35**	.06	.52**				
5. Subjective norms	9	.86	2.99 (.74)	− .03	− .03	.04	.01			
6. Perceived discomfort	5	.69	1.74 (.66)	− .27**	− .05	− .49**	− .35**	− .02		
7. Perceived lack of information	2	.76	2.13 (1.01)	− .31**	− .13**	− .49**	− .35**	− .13**	.38**	
8. Behavioural intentions	7	.90	4.53 (.47)	.38**	.11*	.62**	.45**	.15**	− .49**	− .52**

*M* mean, *SD* standard deviation

* *p* < .01; ** *p* < .001

### Malaria preventive measures

**Fig. 3:**
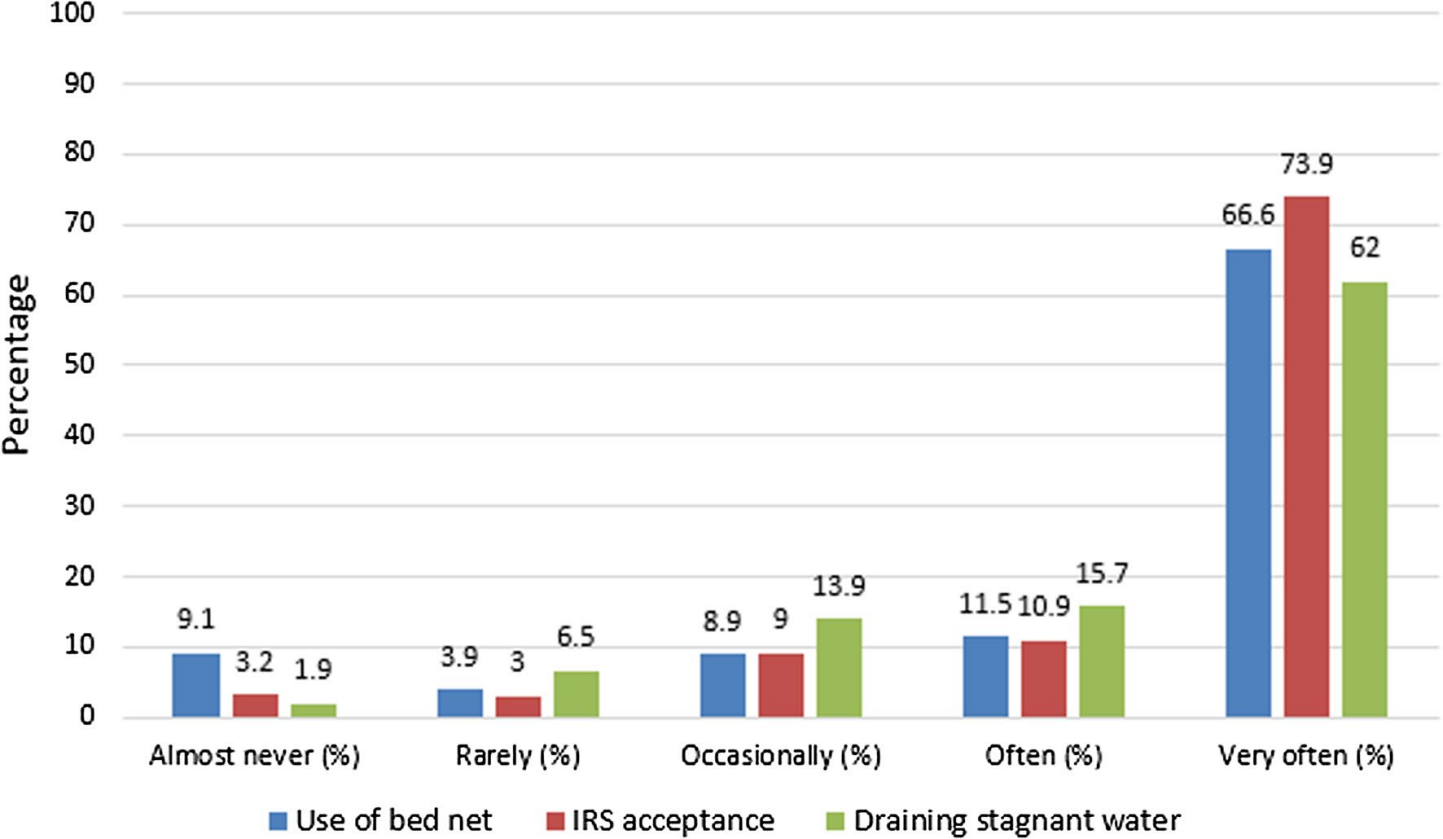
Use and acceptance of malaria preventive measures. This figure shows how the study participants perform three different malaria preventive measures (LLINs, IRS, and draining of stagnant water) using a five point Likert scale

Respondents were asked to rate the frequency of performing three different malaria preventive measures. The results revealed that 66.6% of the participants very often (consistently) uses an LLIN, 73.9% accepts IRS to be done in their house, and 62.0% drains stagnant water in case it is present. No apparent substantial differences across the three malaria preventive measures studied was found (see Fig. 3).

### Predictors of behavioural intentions to use malaria preventive measures

The results show that perceived severity, perceived self-efficacy, perceived response efficacy, and subjective norms were positively related to behavioural intentions, while perceived discomfort and lack of information were negatively related. The full model explained 50% of variance of behavioural intentions (see Table 3). Looking at the control variables, there was no significant difference in behavioural intentions between male and female, and between older and younger participants. However, there is a slight significant positive relationship between behavioural intentions and education.

**Table 3:** Regression analysis of predictors of behavioural intentions to use the malaria preventive measures

Steps	Variables	Behavioural intentions	
		1	2
1	Age	.03	.00
	Gender	.02	.00
	Education	.08*	.01
2	Perceived severity		.09**
	Perceived susceptibility		.01
	Perceived self-efficacy		.31***
	Perceived response efficacy		.11***
	Subjective norms		.11***
	Perceived discomfort		− .19***
	Perceived lack of information		− .20***
	R^2^ change		.50***
	Adjusted R^2^	.00	.50***

Standardized regression coefficients are reported

* *p* < .05; ** *p* < .01; *** *p* < .001

### Moderation analysis of behavioural intentions and use of malaria preventive measures

Interaction terms were computed from availability and accessibility of LLINs and behavioural intentions. The results showed that behavioural intentions were positively related to ITNs use, IRS acceptance, and draining stagnant water in case present. However, no evidence supported the hypotheses that either availability or accessibility of LLINs moderated the relationship between behavioural intentions and actual use of LLINs (see Table 4). Looking at the control variables, a slight significant associations between gender, education and use of LLINs, and a significant positive relationship between age and draining stagnant water were observed.

**Table 4:** Moderation analysis on behavioural intentions and use of malaria preventive measures

Steps	Variables	LLINs			IRS		Stagnant water	
		1	2	3	1	2	1	2
1	Age	− .02	− .05	− .04	− .00	− .01	.26*	.22*
	Gender	− .08	− .08	− .08*	.04	.03	− .02	− .04
	Education	.09*	.06	.06	.01	− .00	.05	.04
2	Behavioural intentions		.10*	.10*		.22***		.21*
	Availability		− .02	− .01				
	Accessibility		.08	.07				
3	Interaction term 1 (behavioural intentions and availability)			− .05				
	Interaction term 2 (behavioural intentions and accessibility)			.07				
	Adjusted R^2^	.01*	.02*	.02	− .00	.04***	.03	.07*

Standardized regression coefficients are reported

* *p* < .05; *** *p* < .001

**Fig. 4:**
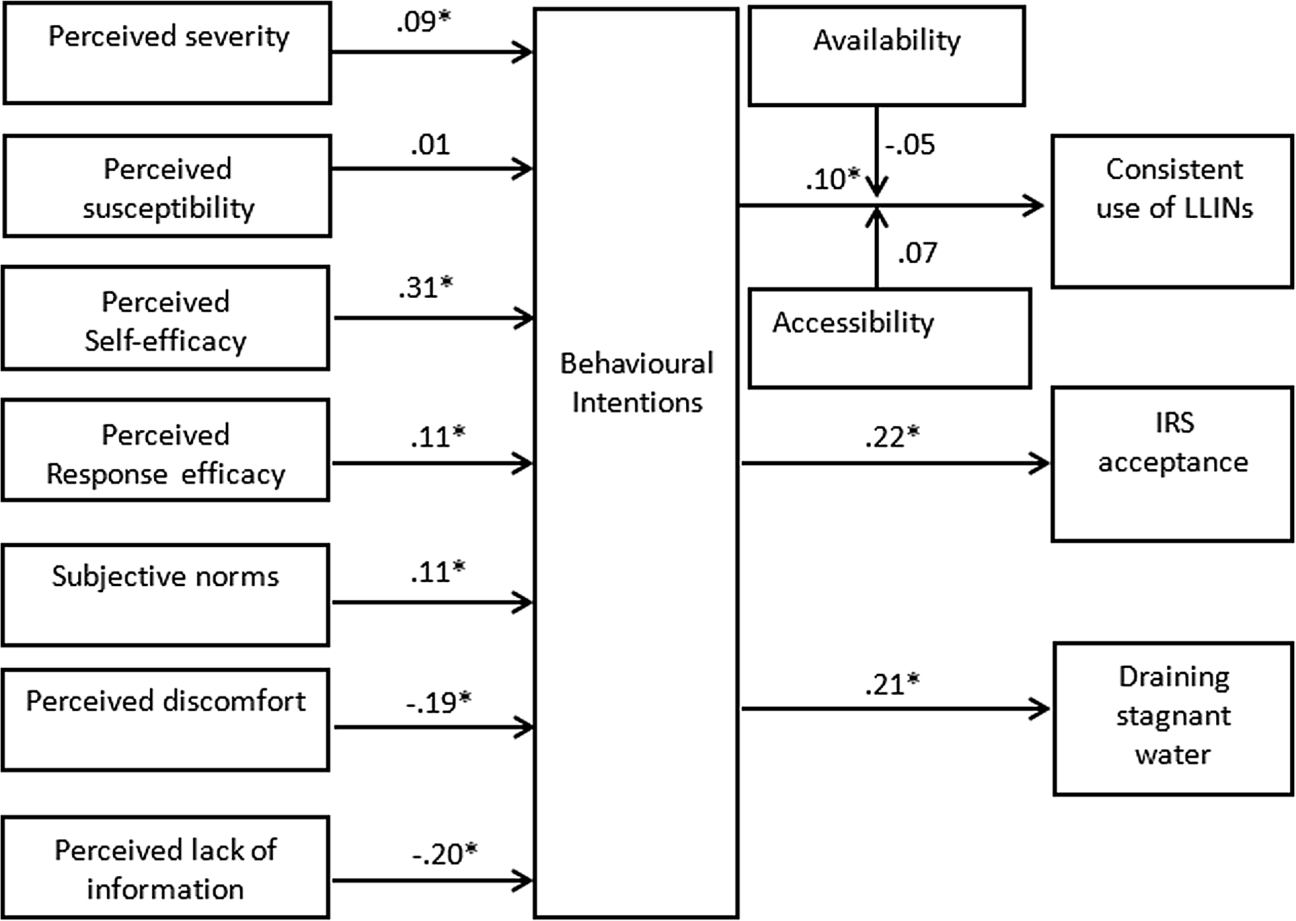
Results of the integrated model showing the relationships (* indicates a significant relationship). This figure shows the summary of the results from testing the conceptual model. It points out how perceived severity of malaria, perceived self-efficacy, perceived effectiveness of malaria preventive measures, subjective norms, and perceived barriers (perceived discomfort and lack of information) influence intentions to use malaria preventive measures. In addition it indicates the results of moderation analysis of both availability and accessibility between intentions and actual use of LLINs; and the relationship between behavioural intentions and actual use of LLINs, acceptance of IRS, and draining of stagnant water

In summary, intentions to use malaria preventive measures were positively influenced by perceived severity of malaria, perceived self-efficacy, perceived effectiveness of malaria preventive measures, and subjective norms, and negatively associated with perceived barriers (perceived discomfort and lack of information). There was no significant evidence supporting the moderation between either availability or accessibility of LLINs to behavioural intentions and use of LLINs (see Fig. 4). Standardized coefficients are reported.

### Phase two

Based on the relationships that were found in the quantitative survey, a more in-depth understanding of the mechanisms that explain these relationships was obtained. This was done by exploring different perceptions related to malaria and malaria preventive measures, and identifying strategies to enhance the consistent use of these measures. Therefore, phase two employed focus group discussions and interviews to gain this in-depth understanding.

## Methods

### Selection of participants

A total of 76 residents of the Ruhuha sector took part in nine Focus Group Discussions (FGDs), seven Key Informant Interviews (KIIs) and three in-depth interviews (IDIs). A homogenous purposive sampling method was used to select the community members to participate in three FGDs of male, female, and youth respectively. The selection of participants with similar characteristics (age and gender) in FGDs was performed to ensure that all participants had equal opportunities to share their views. Consequently, male, female, and youth groups were selected. Other groups were identified based on a stakeholder analysis described in a previous study [26]. The FGDs were composed of (1) one group of Community Health Workers (CHWs), (2) one group of members of Community Malaria Action Teams (CMATs), (3) one group of female community members, (4) one group of male community members, (5) one group of youth community members, (6) three groups of cooperative members, and (7) one group of religious leaders. Initially, the target was to have eight participants per group discussion, however, on the scheduled day, some participants were unable to attend the discussion due to different personal reasons. Consequently, the youth and male groups had seven participants each and the religious group had five participants. Initially, ten FGDs, ten KIIs, and three IDIs were scheduled to start with. Following the review and assessment of the transcripts and notes taken, after conducting nine FGDs, seven KIIs, and three IDIs, saturation had been achieved as no new data was appearing and all concepts in the conceptual framework were fully developed.

At the start, contact details of suitable CHWs and CMATs that could be contacted for FGDs and interviews were obtained from the local health centre. Male, female and youth community members’ participants were identified by the CHWs and CMATs. Cooperative members and church leaders were selected with the help of Ruhuha sector staff. In addition, the interviews with sector level staff and policymakers were scheduled by the researcher. Participants from KII included the representative of CHWs at cell and sector level, in charge of cooperative’s union, one participant at the sector level, and one participant at the national level. The participants of IDI involved three community members (a male, female, and a young person, aged around 20 years).

### Data collection

A focus group guide and interview guide (one for IDI and another for KII) were developed based on concepts from the conceptual framework (see Fig. 1) and the research questions. Minor adjustments and few probes were added throughout the data collection based on previous FGDs, and interviews. Data were collected by one PhD researcher trained in qualitative data collection. A digital voice recorder was used alongside with taking notes. Verbal consent for participation and recording was obtained prior to the start of each FGD and interview. Only one interview participant refused to be recorded. In that case, the researcher only took notes. The FGD lasted between 70 and 90 min, while both IDI and KII lasted approximately the same time and took between 40 and 60 min. The interviews with sector level staff and policymakers were conducted in their offices or at another suitable location. The remaining interviews were conducted at the health centre. The language used during all discussions and interviews was Kinyarwanda as all participants were proficient in this language.

### Data analysis

Thematic analysis followed by a mainly deductive method was employed to analyse the data [27, 28]. Data were evaluated following the consolidated criteria for reporting qualitative research (COREQ) guidelines [29]. The integrated model of determinants of malaria preventive behaviour guided the analysis and its concepts served as the main themes. However, in case additional themes emerged, then those were added. Voice recorded interviews and FGDs were transcribed and translated into English. Transcripts were verified for completeness and checked to ensure that personal identifiers were deleted. The research team conducted an iterative revision and discussion about verbatim texts. Analysis of the text resulted in an initial set of categories that were independently developed by the first author. This was done following the deductive nature of content analysis, which was driven by a predefined conceptual framework and related concepts, which were considered the main themes. Other members of the research team (2nd, 3rd and 4th authors) further independently reviewed this initial set of coding and suggested additional categories to increase the readability of the findings. These categories were further matched with the list of corresponding themes. As not much discrepancy was observed and in case discrepancy in the categories was found, then this was resolved before they were applied to quotes. Hence, inter-rater agreement and kappa were not calculated as was not deemed necessary. In presenting the results, the headings reflect the main themes used for the analysis and subheadings reflect the categories that emerged during the discussion and the process of reading and coding the transcripts.

## Results

In this section, the results are reported based on the seven themes of discussion: (1) perceived severity of malaria, (2) perceived susceptibility to malaria, (3) perceived self-efficacy to use bed nets, (4) perceived response efficacy of bed nets, (5) perceived barriers to use bed nets, (6) IRS acceptance, and (7) strategies to enhance the consistent use of malaria preventive measures. As shown in the sections below, some of these themes indicated more variation among participants compared to others, hence these are reported using different categories that emerged during the discussion.

### Perceived severity of malaria

Participants indicated that the incidence of malaria clearly increased in 2017 relative to 2016. Malaria severity and an increase in the number of malaria cases was discussed and four categories emerged: (1) malaria is perceived as an epidemic, (2) a high number of malaria cases in relation to high mosquito density, (3) increased malaria cases in relation to weather as well as (4) repetitive episodes of malaria in relation to low perceived effectiveness of Coartem^®^ (brand name of artemether–lumefantrine, one of the medicines recommended for artemisinin-based combination therapy).

#### Malaria is perceived as an epidemic

Malaria was widely believed to be a serious disease and more severe than it used to be in the past (in 2017 compared to 2016). With no exception, participants perceived that malaria posed a very real threat. They expressed worry as most of the household members suffer from malaria from time to time and it is hard to find a family without a malaria case:

“*In the past, malaria was not considered as a severe disease, however nowadays it has become an epidemic. When you visit one household, you find that, for example, five members suffer from malaria. For this reason, people are even telling us to ask the health professionals on their behalf whether this is malaria as it used to be or whether it is an epidemic.” (CHWs FGD)*

*“Nowadays people are asking themselves what kind of disease is this? 2017 came with a difference in relation to malaria,, compared to 2016. Even people are calling each other to go to the health centre together as if they are going to pray together!.” (Male FGD)*

#### A high number of malaria cases in relation to high mosquito density

Some participants indicated that there is a high mosquito density and mosquito nuisance. The participants also reported an increase in perceived severity of malaria. Mosquitoes were reported to be everywhere in or outside the house and they can bite anytime especially during the night when sleeping:

“*These days in the evening, you hear a lot of mosquito noises like bee buzzing. There are a lot of mosquitoes these days, and this makes me think that even if you are protected under the bed net at night you can even get a mosquito bite outside the house, and get malaria.” (Female FGD)*

#### Increased malaria cases in relation to weather

Few participants associated the malaria burden with seasonality. In this line, it was perceived that malaria cases increase when it starts raining (in September) as a result of rapid mosquito reproduction, and malaria cases decrease during the dry season (in July):

*“Because of change in weather so many people are being affected. Nowadays, we are in a short rainy season (locally termed* Umuhindo*), and we know that there is a lot of multiplication of mosquitoes in this period. Although mosquitoes multiply throughout all seasons, this can be one of the reasons for this increase of malaria cases. “(Cooperative FGD)*

#### Repetitive episodes of malaria in relation to the low perceived effectiveness of Coartem^®^

By having repetitive malaria episodes without cure, respondents reported that these episodes may be due to low effectiveness of the malaria medicine, and reported a need to either replace the current medicines and bring ones with high effectiveness or find out a malaria vaccine. Participants believed that normally when you have malaria and get treated, one cure of Coartem^®^ is enough for the sick person to get better. However, their experience is that this is no longer the case. Some of the participants reported to prefer buying some medicines in private pharmacies and perceived that they have a higher quality than those found at the health centre:

“*Malaria has increased this year (2017), there are many malaria cases. We have observed that the medicines that used to cure malaria patients, now it seems like they are not curing. These days a person completes the cure without getting better, and after some days a person falls sick again. Thus, personally, I see that the current medicines may no longer have a strong capacity to cure malaria.”(KII Female participant)*

*“When you discuss with people, they tell you that when they go to the health centre and get medication, they don’t get cured, but when they go to the private pharmacy and buy medications [there are some medications for 3500 Rwf, approximately 4 US dollars] they get cured. Why this, they asked? Do the medicines from the health centre have the same capacity to treat malaria as those at the pharmacy?” (Youth FGD)*

### Perceived susceptibility to malaria

There was a general agreement about susceptibility to malaria among the participants and there was not much variation in the responses indicated. Everybody was considered at risk of getting malaria, and this was mainly due to the observed increase in malaria prevalence. Participants reported that there are many people who are going for treatment and that during the last years they did not suffer from malaria, but now they got it. There is a diversity in those people affected and you can not say that it is only one group of people or certain age group, rather all people, men, women, and children:

*“This year was so special to me. I have never suffered from malaria before, but some months ago I got it and it was serious. I went to the CHW and had it diagnosed. After a few days, my wife and children also had it. Now, being either a child, young adult, or old person does not matter. Everybody is getting malaria.” (Male FGD)*

*“Last time I treated an old woman (81* *years) and she told me that it was her first time to suffer from malaria in her life. She told me that it is an epidemic and not malaria as such. She was advising me to go and ask the health professionals about this epidemic in [cell name was removed] this cell.”(CHWs FGD)*

### Perceived self-efficacy to use a bed net

There was not much variation in the reported self-efficacy to use a bed net. All participants agreed that community members are confident in their ability to use a bed net when available. They reported that sleeping under a bed net is an activity that they are confident to perform. In addition, they also mentioned that it is their responsibility to monitor their children (mostly children under 5 years of age, as these cannot put the bed net on the bed themselves) so that they are sure that they sleep under the bed net once they own it:

*“Sleeping under a bed net does not cost anything and I believe that even for those who don’t have a bed frame it is not a problem, they hang the bed net on the wall and it looks fine.” (CHWs FGD).*

### Perceived response efficacy of LLINs

Many participants believed LLIN to be an effective measure to prevent malaria. Although this was reported, many participants indicated the observed high-perceived effectiveness of LLINs to be related to the increase of malaria incidence (reported as an increased number of malaria cases), and the reduction of the number of bed nets owned. This reported effectiveness of LLINs was extended beyond malaria prevention as some participants highlighted the non-malaria benefits of the LLINs. However, few participants noted that the observed increase of malaria incidence is beyond the capacity of LLINs protection. The reported perceived effectiveness of LLINs could be split into three categories: (i) increase of malaria incidence in relation to the decrease in the number of LLINs owned; (ii) non-malaria benefits of LLINs; and (iii) perceived absence of insecticide and type of LLINs owned.

#### The increase of malaria incidence in relation to the decrease in the number of LLINs owned

It was clear that the increase of malaria cases and associated consequences is related to the reported high-perceived effectiveness of bed nets. Given the high malaria incidence, participants reported that even when it is too hot to use bed nets, the nets are an important tool in preventing being bitten by mosquitoes including those causing malaria. The reported effectiveness is also linked to the decrease of LLINs ownership as many participants reported planning to use them, and, unfortunately, they do not own them anymore as some of the participants noted that the LLINs received from last distribution were used for other unintended purposes:

*“Since malaria severity has gone up people started appreciating the importance of the bed nets. Unfortunately, some of them don’t have the bed nets as from the last distribution some people received the bed nets but they used them for other purposes.” (KII Male participant)*

*“During the previous years, people did not value the bed nets and there weren’t many malaria cases. However, nowadays as malaria cases have increased, they started giving them value when they don’t have them. It is similar to the other proverb “utaribwa ntakinga” meaning that you increase awareness when you lose.” (FGD CHWs)*

#### Non-malaria benefits of LLINs

Apart from being a malaria preventive measure, protection against other insects or reptiles that may fall from the roof while sleeping were reported as non malaria related benefits of LLINs. Some of the participants highlighted that sleeping under a LLINs has become a common practice. They are considering bed nets as something that protects them without thinking necessarily about mosquitoes. Even if it can be hot, they prefer to sleep under the bed net and leave aside the bed sheets:

*“I have never slept without a bed net because it can even protect me from other insects or reptiles like lizards that may fall over. For example, I was sleeping one day and I started feeling sand/pebble falling from the roof. When I looked, it was a snake climbing the wall. Therefore, if I had not slept under the bed net, it would have bitten me. Thus, sleeping under the bed net and making sure you insert it properly under the mattress protects you from so many things.” (CHWs FGD)*

#### Perceived absence of insecticide and type of LLINs owned

Due to high-perceived severity of malaria, some community members believe that this severity of malaria exceeds the capacity of the LLINs. People believe that the LLINs do not have the proper insecticidal protection, or the insecticides are not effective anymore and the LLINs are no longer able to keep the mosquitoes away. Participants indicated that normally mosquitoes would die immediately when they get into contact with the LLINs, however, that is not the case, rather mosquitoes just remain alive on LLINs. Few participants reported also the large mesh size of some type of LLINs and perceived that they can allow mosquitoes to enter and bite the person while sleeping:

*“A few months ago, I got a new bed net from the health centre, and I currently sleep under it, but my child gets sick very often and sometimes everybody at home is sick, then I am wondering why? I think the bed nets are not effective anymore.” (Male FGD)*

*“I think the main reason why many people are affected by malaria is that these bed nets do not have the insecticide for protection, therefore the mosquitoes can bite anytime. I have also seen that the current bed nets have big mesh and I believe that they can even allow mosquitoes to enter and bite the person sleeping under it. Truly they are not protecting us.” (Female FGD)*

### Perceived barriers to use LLINs

Participants indicated three types of barriers that affect the use of LLINs. This can be thematically grouped under: (i) lack of or limited availability of LLINs; (ii) discomfort due to hotness, irritation, and bed bugs; as well as (iii) weak malaria risk perception.

#### Lack of or limited availability of LLINs

Lack of bed nets was mentioned by most of the participants to be the first barrier to use them and automatically put them at risk of getting malaria. Even for LLINs that are still intact, participants mentioned that they no longer contain insecticidal effects which underscore the need for replacement or retreatment (a procedure that was done by using “*Karishya*” kits that could be found in different shops, but they are no longer available). However, with the LLINs, retreatment is no longer necessary as they are treated with insecticides in the factory and this eliminates the needs for retreatment. Participants highlighted that the LLINs are only available for those attending antenatal consultation or vaccination:

“*These days, people don’t have bed nets. Most of the households do not have bed nets. It is even clear because once an infected malaria mosquito bites somebody in the family, you will see that after some time all family members are sick because of lack of preventive measures. This means that the same malaria mosquito stays in the house and bites all of them.*” *(CHWs FGD)*

*“These days people get bed nets at the health centre from antenatal consultation or child vaccination at 9* *months. Those who don’t attend consultation or vaccination services have a problem of getting bed nets.” (Male FGD)*

#### Discomfort due to hotness, irritation, and bed bugs

Discomfort while sleeping under LLINs in the dry season, irritation (especially for those using the bed net with a strong texture or getting in contact with LLINs when using it for the first time), and presence of bed bugs while sleeping under the bed net were reported as hindrances to use bed nets. Many participants noted that the discomfort used to be the main hindrance. However, it was reported by few participants only, as perceived malaria risk exceeds the reported discomfort and many people have changed their opinion/perception:

“*There is a time you go around mobilizing people to sleep under the bed net, but some, honestly tell you that if they use a bed net, they get irritated. And these days many people have bed bugs in their beds which prevent people to use bed nets even if they own the nets.” (KII Female participant)*

*“In my house, I have not moved from my sleeping room because of a mosquito, but I have moved because of bed bugs. It is really annoying. Therefore I pay much attention to the bed bugs more than I do for the mosquitoes and I can not sleep in a bed net when bed bugs are there.*” *(Male FGD)*

*“Due to the hotness that is here in Bugesera, sleeping under the bed net sometimes is like a punishment. And you may say that “even if I get a mosquito bite and get malaria, I will get treated instead of sleeping the whole week in this condition, it is really very hot.”(KII Male participant)*

#### Weak malaria risk perception

Although some respondents mentioned bed bugs as a barrier to sleep under a bed net, others believe that more attention should be on mosquitoes rather than bed bugs. This was explained in relation to the consequences of malaria where also the cost of treatment is involved once the person gets sick. Participants added that when a person is sick, his/her routine activities are disrupted, hence his/her economy is affected. Hence, the perceived malaria risk outweighs the perceived beg bugs’ bites:

*“If there is a room in my house that I can move in and not get into contact with the bed bugs, but no room I can go in and say that I am safe from the mosquitoes, then I should be worried. I know bed bugs are bad and when they bite they cause itching, but after all, I get up in the morning and go to my farms without any problem. In contrast, when an infected mosquito bites me, and I get malaria, in the morning I have to look for somebody to take me to the health centre as I cannot reach there alone. Consequently, this affects my economy”(Male FGD).*

### IRS acceptance

Many of the participants reported the effectiveness of IRS to be good. Participants generally agreed that, after spraying, a person could observe that mosquitoes and other insects were dead. At that time, one could enter in the house without fear as there is no way to get into contact with mosquitoes. However, changing the spraying company was reported to be a problem by the participants. The last spraying activity was done by the Inkeragutabara group (*members of the District Administration Security Support)*, whereas the CHWs used to be responsible for spraying. Therefore, this new group was seen as an outsider in the community and participants highlighted that they did not spray properly. Participants believed that the sprayers had diluted the insecticide, or they are simply not well trained and qualified for the spraying activity. Thus, people were reluctant to accept IRS in their houses:

*“CHWs used to be the one spraying, and to be honest the community members have very much trust in CHWs. But last time the people called “Inkeragutabara” were selected to do the spraying activities. Even some households were not sprayed. We don’t know whether they were entering the house and remain there until getting out without spraying.” (CMATs FGD)*

*“People are willing to receive the sprayers, but the main problem is those sprayers who don’t spray properly. The last team came to spray, but really, they were not spraying, because even at the end of the day (after spraying) you could see the mosquitoes flying. Therefore, some people were hesitant to stay at home and wait for the sprayers as people think that they are wasting their time, waiting for those people for nothing, and they close and go in their farms.” (Female FGD)*

### Strategies to enhance the consistent use and acceptance of malaria preventive measures

A number of strategies to enhance the consistent use and acceptance of malaria preventive measures was mentioned by the participants. These were classified into three categories: (i) availability of LLINs and regular spraying of insecticide; (ii) community mobilization; and (iii) citizen engagement in malaria preventive activities.

#### Accessibility of LLINs and regular spraying of insecticide

Making LLINs available, and regular spraying of insecticides both in houses and in marshlands were reported to be strategies to increase consistent use and acceptance of these measures. For LLINs, participants reported that if they were available in local shops for a cheaper price, then they would buy them. However, others indicated that there are those who cannot buy them either because they cannot afford them or simply because they used to get them from the government for free and believe that they cannot spend money buying the LLINs:

*“Even if the bed net cannot be freely distributed, at least they can be put in local shops and at cheap prices for us to be able to buy them. But you need to have a place where you can buy it. For example in my family, I only have two bed nets and I need three more, therefore, if there is a place to buy, definitely I will buy them unless they are too expensive.” (KII Male participant)*

#### Community mobilization

Community mobilization was generally highlighted as a strategy to promote the consistent use of malaria preventive measures. The focus should be more on those who do not use or accept the malaria preventive measures. Mobilization can be done through monthly community work, *akagoroba k’ ababyeyi* (parents’ evening meeting), and *isibo* meetings (roughly 15 households neighbouring each other), and home visits. Mobilization should be the responsibility of every community member in collaboration with CHWs:

*“We need to continue community mobilization. I know people hear mosquitoes when they make much noise, especially in the rainy season. So, we need to tell them that even in the dry season mosquitoes are there and they have to use the preventive measures consistently even when having bed bugs.” (IDI Youth participant)*

*“Community mobilization related to malaria severity, its consequences, and benefits of bed net use is still lacking. This can be done in a formal and informal meeting at the amasibo, village, and cell levels. Community work and parents’ evening meetings also are good opportunities to share this type of information and mobilize people to remove all kinds of mosquito breeding sites that can be around their homes. Those who never attend the meetings can be visited at their home.” (Youth FGD)*

#### Citizen engagement

As everybody is at risk of getting malaria, participants noted that every citizen should be actively involved in malaria prevention activities. When everybody feels responsible and actively contributes to malaria prevention through using LLINs consistently, controlling mosquito breeding sites, and accepting IRS, then mobilization could be easier. Thus, proactive mechanisms, discussions, and interactions between community members can be enhanced:

*“Every community member should feel it [malaria prevention and control] as a personal responsibility, because even if you can mobilize, but people don’t feel responsible, then nothing can be changed. But if everybody engages in malaria prevention, I believe that perceptions can be improved.” (Youth FGD)*

*“Even if you distribute the bed nets today, tomorrow you will not find them as long as the perceived discomfort and bed bugs are still there. Do you know what they are saying? They are saying that the bed bugs go to the bed net in the night. In their feeling, they say that bed nets bring bed bugs. They remove them and burn them. Consequently, even if you distribute bed nets today, you will not solve the problem. First of all, let’s target the issue of bed bugs and related perceptions, and then we distribute the bed nets later.” (KII female participant)*

## Discussion

This study used a mixed methods approach to assess the relationship between individual perceptions and intentions to use malaria preventive measures, to explore why people have certain perceptions, and to identify strategies that stimulate consistent use of malaria preventive measures.

### Perceived severity and susceptibility

A high-perceived severity of malaria among the study participants was reported and this was found to have a significant positive association with intentions to use malaria preventive measures. High-perceived malaria risk in relation to mosquito density was also reported elsewhere [23, 30]. Watanabe et al. [19] found a weak malaria risk perception to be associated with a reduction in malaria incidence and disappearance of mosquitoes in the dry season. Thus, a high-perceived severity of malaria reported in present study could be attributed to the time of the study (short rainy season) in which mosquito density is expected to be high, consequently people intend to use LLINs to prevent mosquito nuisance.

Regarding susceptibility, Beer et al. [23] reported children to have a greater chance of contracting malaria than any other group of the population. The reported high-perceived susceptibility among the majority of the study participants in the current study was due to high-perceived severity of malaria and to repetitive malaria episodes among the majority of the community members. The perceived low effectiveness of Coartem^®^ also increases the perceived severity of malaria as well susceptibility. Artemisinin-based combination therapy is considered the first line treatment and the most effective anti-malarial drug [1, 3]. The perceived low effectiveness of Coartem^®^ is challenging as it may lead to possible incorrect self-treatment through buying medicines in the pharmacy without a diagnosis, hence drug resistance in the longer term. While the reasons for lack of a significant relationship between perceived susceptibility and intentions to use malaria preventive measures are not fully clear, however, this may be due to the fact that people who live in malaria-endemic areas are familiar with the malaria risk, hence become more accustomed to it.

### Perceived self-efficacy and response efficacy

The finding that the perceived response efficacy is related to the observed increase of malaria cases is consistent with previous studies [20, 23, 30, 31]. This indicates that participants are worried about malaria consequences and tend to think about the benefits of malaria preventive measures, hence this may increase their use. In addition, this study revealed a positive association between both perceived self-efficacy and response efficacy, and intentions to use malaria preventive measures. Birhanu et al. [17] showed that in the dry season, when perceived mosquito nuisance decreases, use of LLINs also decreases. It is often an issue in the dry season as people are less concerned about malaria and mosquito nuisance, thus, intentions to consistently use LLINs also decreases. The non-malaria related benefits of LLINs, including avoiding biting insects and a good night sleep were also reported as factors that influence people to sleep under LLINs in Kenya [31], Zanzibar and Tanzania [20, 30], and Vanuatu Islands [19]. Comfortability and provision of warmth during cold weather were also reported in previous studies [20, 30]. This shows that the non-malaria related benefits of LLINs exist and may also be promoted in malaria-related messages, especially in a dry season when the use of LLINs reduces due to the decrease of malaria incidence and mosquito density.

While some criticized the type of the bed nets (strong texture and large mesh size of the net), Beer et al. [23] found that some people prefer them as they do not tear easily and they also allow ventilation. The dissatisfaction about the type and size of bed nets was also reported in a study conducted in Tanzania [30]. If people believe that they can get mosquito bites through the LLINs and get repeated malaria episodes, then they may doubt the effectiveness of LLINs, hence intend not to use them [30]. The perceived lower effectiveness of LLINs was also reported in Ethiopia where participants indicated that the LLINs’ insecticide was unable to kill mosquitoes and other insects, therefore people throw them away or use them for other purposes [17]. Regarding IRS, a high-perceived effectiveness of IRS was reported and behavioural intentions have a positive relationship with IRS acceptance. In contrast, some previous studies have found that IRS acceptability may be more related to the perceived obligation to accept government initiatives rather than based on its effectiveness [14]. The reported high-perceived effectiveness of IRS in this study may be due to the campaigns that have been conducted at the start of spraying activities. Equally, the fact that CHWs also participated in the previous spraying activities may also be an added advantage as they could provide more explanations to some of the households when they refuse to get their houses sprayed.

### Subjective norms and perceived barriers

The result indicates that subjective norms were positively related to behavioural intentions. If many people in the community use and accept malaria preventive measures (LLINs, IRS, and draining stagnant water), then it is more likely that most of the people in the same community will intend to consistently use those measures [5]. However, lack of collective awareness about collective management of, for example, mosquito breeding sites may hinder the plan to consistently use malaria preventive measures [32]. In the same way, collective action that supports the community members to think about malaria preventive measures and related benefits, may influence people to use these measures.

Lack of LLINs was reported to impede the consistent use of LLINs. Other studies also revealed a lack of access to LLINs to be the reason associated with non-use [20, 30]. However, no significant evidence supports that availability or accessibility moderate the relation between intentions and consistent use of LLINs. Thus, regardless of how both availability and accessibility of LLINs are potential factors of LLINs use, we did not find evidence that they are statistically significant moderators in the relation between intentions and consistent use of LLINs. This strongly indicates that when people plan to use LLINs, having them or not may not be an issue. These results are in line with those found by Msellemu et al. [30] who reported that when participants were reminded that they should still be having the LLINs that were distributed freely, accessibility was not an issue anymore, and other measures including closing doors in the evening and use of mosquito sprays were then reported to be preferred over the sleeping under LLINs and participants emphasized the protective value of these measures. The same authors reported that people may spend own money buying LLINs, but still do not use them, and keep them for either visitors or children only [30]. This provides evidence that individual perceptions play a large role in the decisions to use or not use malaria preventive measures and should be addressed prior to or parallel to LLINs distribution and spraying campaigns.

Discomfort, irritability, and bed bugs were reported in previous studies to hinder the consistent use of LLINs [16, 20, 33]. Fear of chemicals in the LLINs that may cause irritation and itching were also cited as reasons for not using LLINs in an Ethiopian study [17]. While reasons for non-use of ITNs were widely reported, it was highlighted in this study that the discomfort of mosquitoes outweighs the discomfort of feeling hot while sleeping under the LLINs or the presence of bed bugs. In the same line with the current study, distrust of household members in sprayers was also reported in other studies to decrease acceptance of IRS [14–16]. In some circumstances, sprayers are recruited exclusively based on where they live, or their religion [14]. Refusal of IRS can be addressed by community mobilization.

### Strategies to enhance the consistent use of malaria preventive measures

It is clear that when a person intends to use the LLINs, then the person can buy them. However, even if the person owns LLINs, and does not see the associated effectiveness, then the person will not use them. In that regard, community mobilization and citizen engagement in the development of malaria prevention strategies is crucial as it promotes acceptability, adherence, and sustainability of those strategies [34, 35]. Community mobilization in form of health education campaigns focusing on the importance of consistent use of LLINs and consequences of not using them was also reported in Tanzania as one of the strategies to increase their consistent use [30]. Considering LLINs as an essential part of life, and becoming accustomed to it, is an important factor and first step of engagement towards maintaining and sustaining the consistent use of them despite fluctuations in perceived malaria risk and malaria incidence [20]. However, mobilization and engagement alone without LLINs distribution in a place where LLINs are limited and even not accessible cannot achieve tangible results [6]. Still, in a place where perceived discomfort can explain non-use of LLINs (e.g. if bed bugs are present) there is no guarantee that the combination of community mobilization and distribution of LLINs would enhance the use of LLINs, rather the engagement and feeling of ownership of these measures should be the first step as it may target and remove or modify the perceived barriers.

Given the public health burden caused by malaria, the following are three important policy and program implications of the findings reported: (1) individual perceptions play a large role in the decisions to use or not use malaria preventive measures and should be addressed prior to or parallel to LLINs distribution and spraying campaigns. This can be done by enhancing and encouraging the feeling of ownership of these measures through community mobilization and citizen engagement in malaria prevention and control activities. In turn this may promote acceptability, adherence, and sustainability of those strategies. (2) Especially in a dry season when the use of LLINs reduces due to the decrease of malaria incidence and mosquito density, non-malaria related benefits of LLINs should be promoted in malaria-related messages organized at national level. Since malaria prevention may not be enough reason for people to consistently use LLINs in dry seasons, other benefits of LLINs should be included in malaria related messages. For example protection against other insects, and provision of a good night sleep should be incorporated in malaria related messages so that sleeping under LLINs consistently becomes a common practice. And (3) for IRS, hiring of sprayers should consider those who are trusted and preferred by the household members (example: CHWs).

## Strengths and limitations

A mixed methods design allowed us to explore the individual perceptions and quantify the effects of different individual perceptions on the intentions to use malaria preventive measures. This is essential as it offers new insights into the field of vector-borne disease risk and malaria prevention in particular. For example, the fact that individual perceptions explain 50% of variance in intentions to use malaria preventive measures suggests that interventions to promote the consistent use of malaria preventive measures should consider adopting an approach based on an integrated model of determinants of malaria preventive behaviour [21]. Use of this model provides a comprehensive understanding of this key area of self-protective behaviour in relation to vector borne diseases. Future research could test this model in other (geographic) settings, in the context of other vector-transmitted diseases, or other behaviour change projects.

The documented reasons of why people have certain perceptions may explain the gap between the ownership and use of LLINs reported in previous studies [5, 8–12]. In addition, the reported lack of a significant evidence supporting the moderation effect of availability of LLINs to intentions and use of LLINs shows that availability and accessibility of LLINs is not enough when people do not have intentions to use them.

While this study provides several useful insights that can be considered when implementing malaria prevention interventions, there may be other factors beyond the individual perceptions that may also play a large role in the consistent use of malaria preventive measures. However, focussing on individual perceptions allows for quantifying how important these perceptions are, and indicates how they should be taken into consideration when designing malaria prevention interventions. Future research should take into account other factors beyond the individual perceptions in order to give insights on how they also influence intentions to use malaria preventive measures. These factors include issues pertaining to collective action.

The study was conducted during the short rainy season (October and November) which is one of the peaks for malaria transmission and, therefore, this could have affected the perceived severity and perceived effectiveness of LLINs. Thus, future studies that explore the variation of individual perceptions across different seasons are warranted.

## Conclusion

The aim of this mixed methods study was to assess the relationships between individual perceptions and the intentions to use malaria preventive measures by applying an integrated model for determinants of malaria preventive behaviour. In addition, the study also explored the viability of strategies that stimulate consistent use of malaria preventive measures. Perceived severity of malaria, self-efficacy and response efficacy of malaria preventive measures, and subjective norms were reported to influence intentions to use malaria preventive measures consistently. Irritation, increase of warmth, and bed bugs were frequently cited as the main reasons for not using LLINs. Although IRS was perceived to be effective, distrust in sprayers affected the acceptance of IRS. While not having LLINs was frequently reported by most of the participants as impediment of consistent use of LLINs, statistical analyses did not support this as the sole factor explaining non-use when have intention. The study also explored whether accessibility can moderate the effect of intentions to the consistent use, and the results did not show any significant moderation effect. The full conceptual model that included individual perceptions explained 50% of variance of behavioural intentions among the participants, and the intentions were significantly associated with the consistent use of LLINs, IRS acceptance, and draining of stagnant water. Thus, future malaria prevention interventions to consistently use malaria preventive measures should consider individual perceptions by taking into account that the intentions are driven by multiple factors at different levels. This can be done by enhancing the feeling of ownership of these measures through community mobilization and citizen engagement in malaria prevention and control activities.

## Data Availability

The datasets used in this study are available from the corresponding author on a reasonable request.

## Abbreviations

CHWs: Community Health Workers
CMATs: Community Malaria Action Teams
FGDs: Focus Group Discussions
IDI: in-depth interviews
IRS: indoor residual spraying
KII: key informant interviews
LLINs: long-lasting insecticide-treated bed nets

## References

[1] WHO (2017). World malaria report.

[2] President’s Malaria Initiative. Rwanda Malaria Operational Plan FY. 2017.

[3] MoH. Revised National Malaria Contingency Plan 2016–2020. Kigali; 2017.

[4] RDHS. Rwanda Demographic and Health Survey 2014-2015. Kigali, 2015.

[5] Babalola S, Adedokun ST, McCartney-Melstad A, Okoh M, Asa S, Tweedie I (2018). Factors associated with caregivers’ consistency of use of bed nets in Nigeria: a multilevel multinomial analysis of survey data. Malar J..

[6] Hetzel MW, Gideon G, Lote N, Makita L, Siba PM, Mueller (2012). Ownership and usage of mosquito nets after four years of large-scale free distribution in Papua New Guinea. Malar J..

[7] Ricotta EE, Boulay M, Ainslie R, Babalola S, Fotheringham M, Koenker H (2015). The use of mediation analysis to assess the effects of a behaviour change communication strategy on bed net ideation and household universal coverage in Tanzania. Malar J..

[8] Ernst KC, Erly S, Adusei C, Bell ML, Kessie DK, Biritwum-Nyarko A (2017). Reported bed net ownership and use in social contacts is associated with uptake of bed nets for malaria prevention in pregnant women in Ghana. Malar J..

[9] Gonahasa S, Maiteki-Sebuguzi C, Rugnao S, Dorsey G, Opigo J, Yeka A (2018). LLIN Evaluation in Uganda Project (LLINEUP): factors associated with ownership and use of long-lasting insecticidal nets in Uganda: a cross-sectional survey of 48 districts. Malar J..

[10] Kateera F, Ingabire CM, Hakizimana E, Rulisa A, Karinda P, Grobusch MP (2015). Long-lasting insecticidal net source, ownership and use in the context of universal coverage: a household survey in eastern Rwanda. Malar J..

[11] Ntuku HM, Ruckstuhl L, Julo-Réminiac JE, Umesumbu SE, Bokota A, Tshefu AK (2017). Long-lasting insecticidal net (LLIN) ownership, use and cost of implementation after a mass distribution campaign in Kasaï Occidental Province, Democratic Republic of Congo. Malar J..

[12] Samadoulougou S, Pearcy M, Yé Y, Kirakoya-Samadoulougou F (2017). Progress in coverage of bed net ownership and use in Burkina Faso 2003–2014: evidence from population-based surveys. Malar J..

[13] Koenker H, Kilian A (2014). Recalculating the Net Use Gap: a Multi-Country Comparison of ITN Use versus ITN Access. PLoS ONE.

[14] Kaufman MR, Rweyemamu D, Koenker H, Macha J (2012). “My children and I will no longer suffer from malaria”: a qualitative study of the acceptance and rejection of indoor residual spraying to prevent malaria in Tanzania. Malar J..

[15] Munguambe K, Pool R, Montgomery C, Bavo C, Nhacolo A, Fiosse L (2011). What drives community adherence to indoor residual spraying (IRS) against malaria in Manhica district, rural Mozambique: a qualitative study. Malar J..

[16] Ingabire MC, Rulisa A, Van Kempen L, Muvunyi C, Koenraadt CJ, Van Vugt M (2015). Factors impeding the acceptability and use of malaria preventive measures: implications for malaria elimination in eastern Rwanda. Malar J..

[17] Birhanu Z, Abebe L, Sudhakar M, Dissanayake G, Yihdego Y, Alemayehu G (2015). Access to and use gaps of insecticide-treated nets among communities in Jimma Zone, southwestern Ethiopia: baseline results from malaria education interventions. BMC Public Health..

[18] Hung WS, Hu SC, Hsu YC, Chen KL, Chen KH, Yu MC (2014). Factors affecting the use of anti-malaria preventive measures among Taiwan immigrants returning to malaria-endemic regions. Travel Med Infect Dis..

[19] Watanabe N, Kaneko A, Yamar S, Leodoro H, Taleo G, Tanihata T (2014). Determinants of the use of insecticide-treated bed nets on islands of pre- and post-malaria elimination: an application of the health belief model in Vanuatu. Malar J..

[20] Koenker MH, Loll D, Rweyemamu D, Ali SA (2013). A good night’s sleep and the habit of net use: perceptions of risk and reasons for bed net use in Bukoba and Zanzibar. Malar J..

[21] Asingizwe D, Poortvliet PM, Koenraadt CJM, Van Vliet AJH, Murindahabi MM, Ingabire C (2018). Applying citizen science for malaria prevention in Rwanda: an integrated conceptual framework. NJAS Wageningen Journal of Life Sciences.

[22] Ankomah A, Adebayo SB, Arogundade ED, Anyanti J, Nwokolo E, Ladipo O (2012). Determinants of insecticide-treated net ownership and utilization among pregnant women in Nigeria. BMC Public Health..

[23] Beer N, Ali AS, Eskilsson H, Jansson A, Abdul-Kadir FM, Rotllant-Estelrich G (2012). A qualitative study on caretakers’ perceived need of bed-nets after reduced malaria transmission in Zanzibar, Tanzania. BMC Public Health..

[24] Kateera F, Ingabire CM, Hakizimana E, Kalinda P, Mens PF, Grobusch MP (2015). Malaria, anaemia and under-nutrition: three frequently co-existing conditions among preschool children in rural Rwanda. Malar J..

[25] MEASURE Evaluation, MEASURE DHS, President’s Malaria Initiative, Roll Back Malaria Partnership, UNICEF, World Health Organization. Household survey indicators for malaria control. 2013.

[26] Ingabire MC, Kateera F, Hakizimana E, Rulisa A, Borne DVB, Muvunyi (2016). Stakeholder engagement in community-based malaria studies in a defined setting in the Eastern Province. Rwanda. Mediterr J Soc Sci..

[27] Braun V, Clarke V (2006). Using thematic analysis in psychology. Qual Res Psychol.

[28] Elo S, Kyngas H (2008). The qualitative content analysis process. J Adv Nurs..

[29] Tong A, Sainsbury P, Craig J (2007). Consolidated criteria for reporting qualitative research (COREQ): a 32-item checklist for interviews and focus groups. Int J Qual Health Care.

[30] Msellemu D, Shemdoe A, Makungu C, Mlacha Y, Kannady K, Dongus S (2017). The underlying reasons for very high levels of bed net use, and higher malaria infection prevalence among bed net users than non-users in the Tanzanian city of Dar es Salaam: a qualitative study. Malar J..

[31] Dye TD, Apondi R, Lugada ES, Kahn JG, Smith J, Othoro C (2010). “Before we used to get sick all the time”: perceptions of malaria and use of long-lasting insecticide-treated bed nets (LLINs) in a rural Kenyan community. Malar J..

[32] Leeuwis C, Cieslik KJ, Aarts MNC, Dewulf ARPJ, Ludwig F, Werners SE (2018). Reflections on the potential of virtual citizen science platforms to address collective action challenges: lessons and implications for future research. NJAS Wageningen Journal of Life Sciences.

[33] Pulford J, Hetzel WM, Bryant M, Siba MP, Mueller I (2011). Reported reasons for not using a mosquito net when one is available: a review of the published literature. Malar J..

[34] Kajeechiwa L, Thwin MM, Nosten S, Tun SW, Parker D, von Seidlein L (2017). Community engagement for the rapid elimination of malaria: the case of Kayin State, Myanmar. Wellcome Open Res..

[35] Sahan K, Pell C, Smithuis F, Phyo AK, Maung SM, Indrasuta C (2017). Community engagement and the social context of targeted malaria treatment: a qualitative study in Kayin (Karen) State, Myanmar. Malar J..

